# Supplementary material for: Rice 3D chromatin structure correlates with sequence variation and meiotic recombination rate
Source: Commun Biol. 2020 May 12;3:235. doi: 10.1038/s42003-020-0932-2 (PMC7217851; doi:10.1038/s42003-020-0932-2)
Supplement: Supplementary file 1 — Supplementary Information [file 42003_2020_932_MOESM1_ESM.pdf]

Supplementary figures

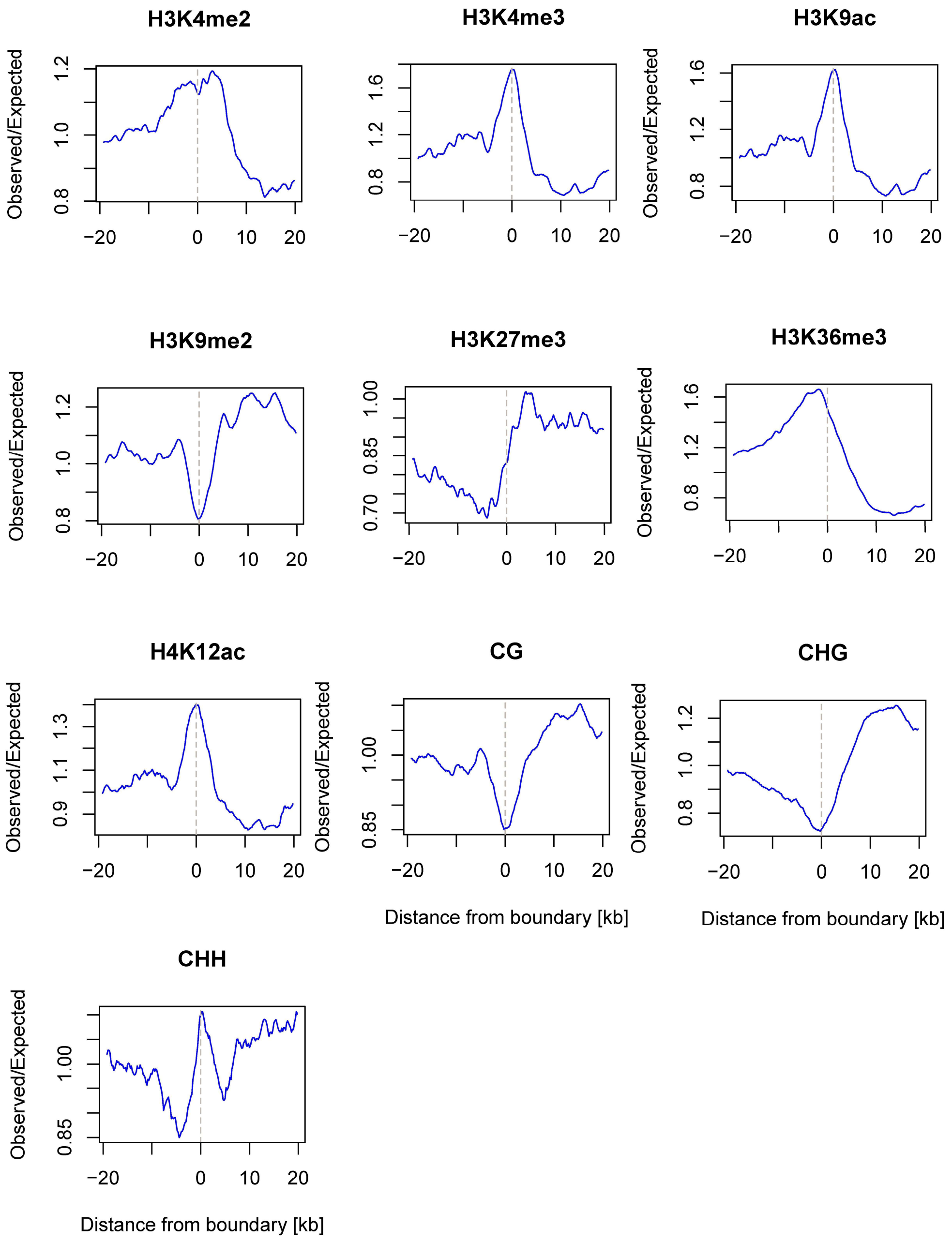

Supplementary Figure 1. Histone and DNA modifications across TAD boundaries.

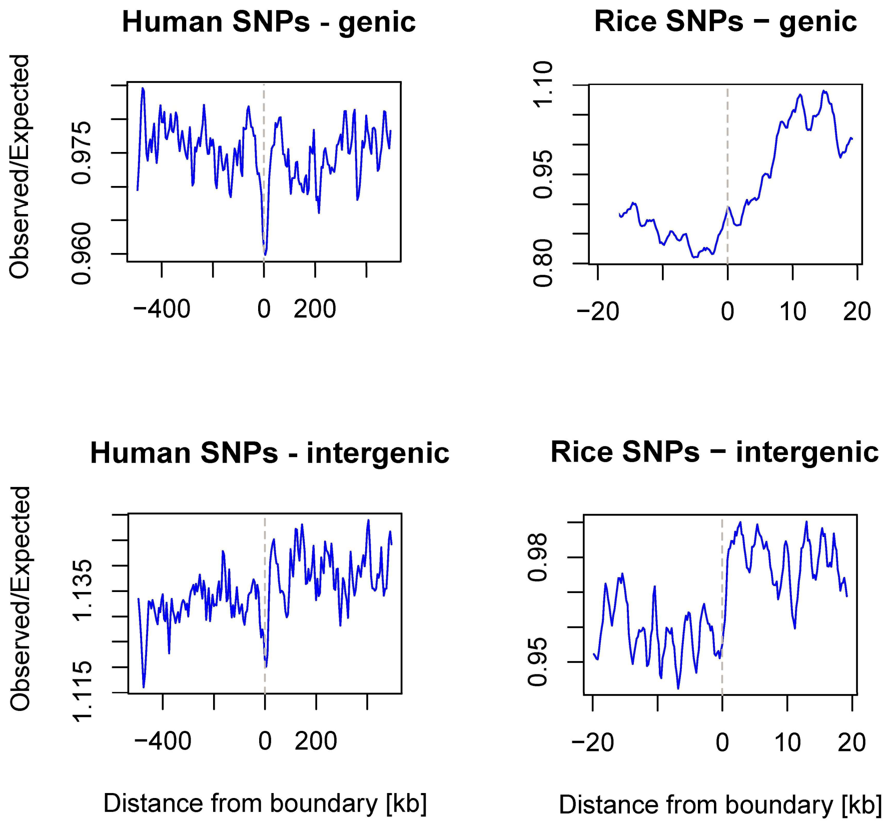

Supplementary Figure 2. Variant profiles across human and rice TAD boundaries. Genic – from the start to the end of gene.

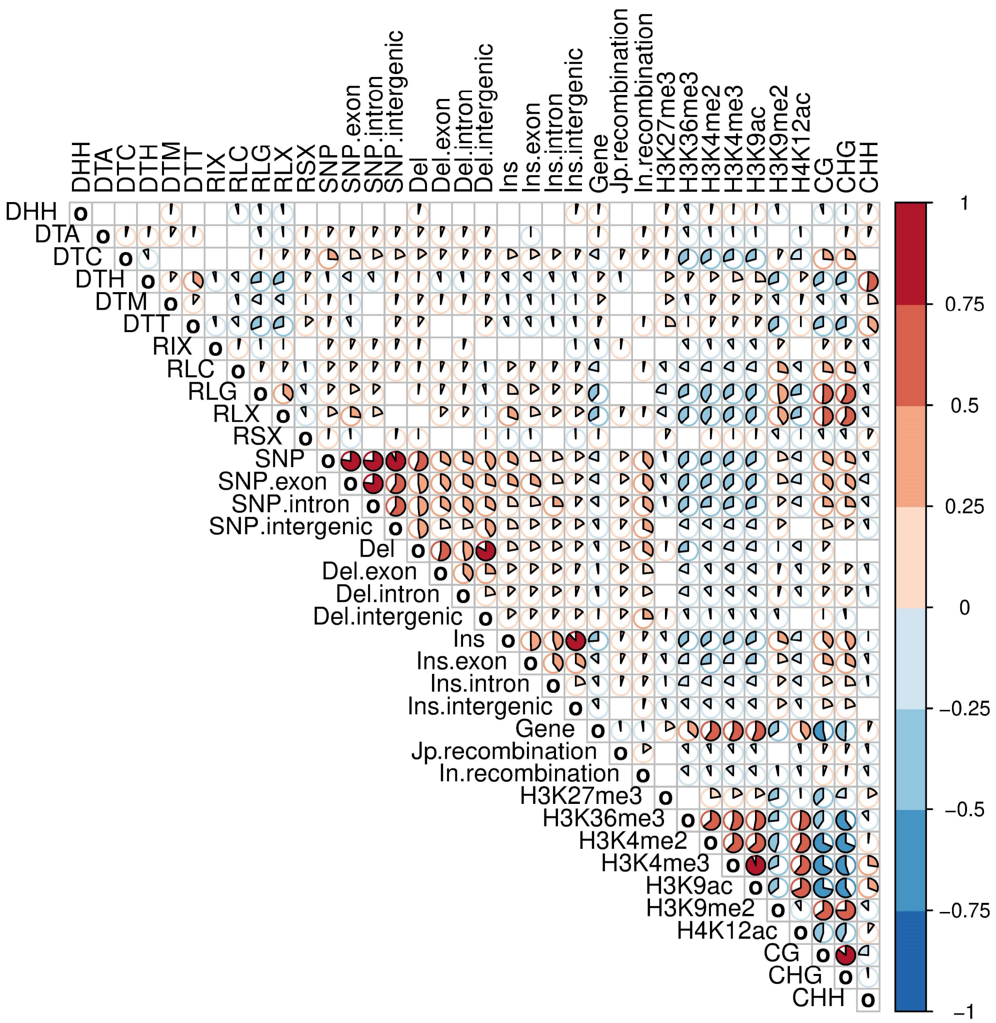

Supplementary Figure 3. Pearson correlations between different genomic and epigenomic features across the rice genome. Non-log-transformed values.

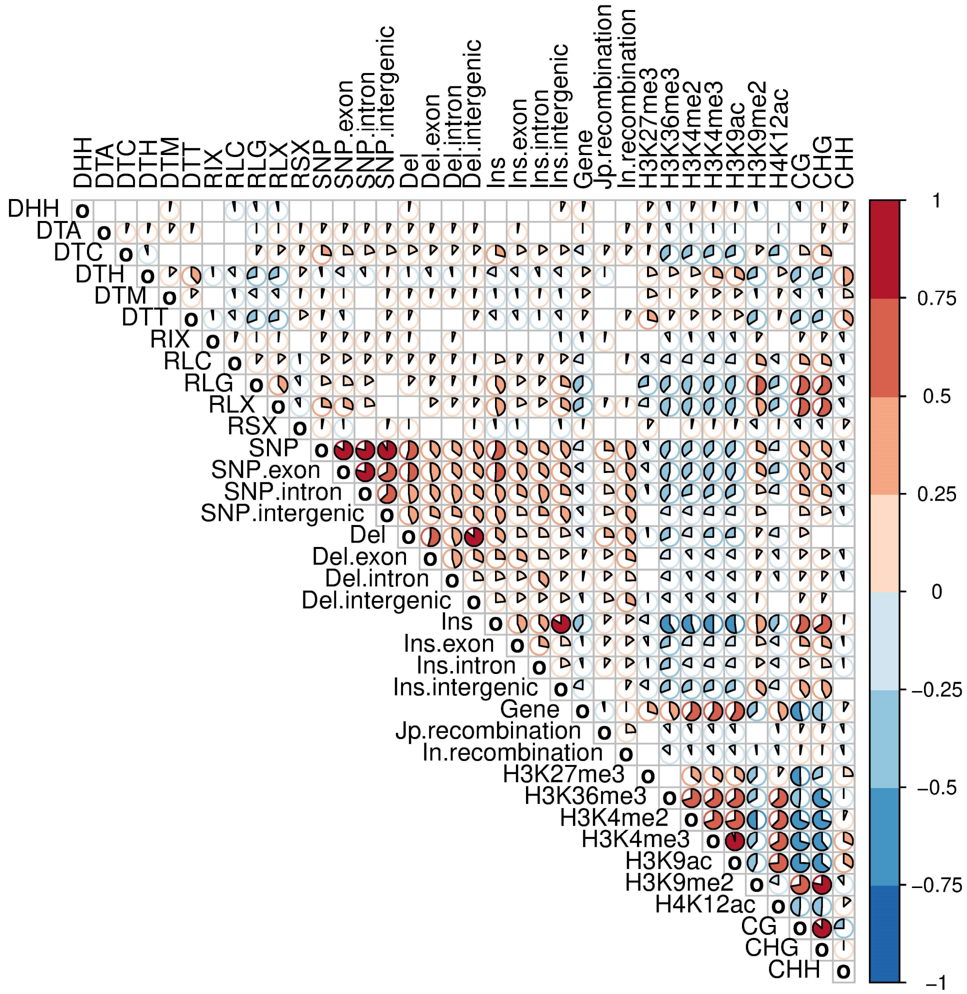

Supplementary Figure 4. Spearman correlations between different genomic and epigenomic features across the rice genome. Non-log-transformed values.

Supplementary tables

Supplementary Table 1. Comparison of the three existing TAD annotations. Note that we used D1 in interaction strength comparisons, as it represents isolated mesophyll signal, not an average across cell types. However, as a result D1 TADs will have an advantage in comparisons as only for D1 the same data was used for TAD calling and interaction analysis.

Dataset alias	Ref.	Median size [bp]	# of domains	Genome coverage [%]	Median interaction (D1 r1+r2)	Mean interaction (D1 r1+r2)
D1	Dong et al.[1]	160,000	1,917	95	6.52	11.5
D2	Liu et al.[2]	45,000	1,763	31.6	10.47	16.44
D3	Dong et al.[3]	450,000	526	68.6	3.37	7.03

Supplementary Table 2. Armatus domain statistics using different values of parameter γ.

γ	Median size [bp]	# of domains	Genome coverage [%]
0.3	40,000	4,075	74.9
0.4	35,000	4,551	68.6
0.5	30,000	4,829	60.6

Supplementary Table 3. Summary of TAD calls using three different datasets. *Number of valid interactions is proportional to sequencing depth which was lowest for D1 and highest for D3 dataset.

	# Valid interactions*	Median TAD size [bp]	# of domains	Genome coverage [%]	Median interaction strength in dataset (r1+r2)			Mean interaction strength in dataset (r1+r2)		
					D1	D2	D3	D1	D2	D3
D1	36,438,978<br>38,816,900	30,000	4,409	56.9	10.2	34.5	47.6	16.7	57.9	67.3
D2	141,236,885<br>149,407,091	35,000	4,599	69.7	7.9	27.8	36.7	14.1	50.6	56.6
D3	247,192,814<br>192,878,099	40,000	3,644	67	5	13.6	20.4	10.7	33.1	40.4

Supplementary note

Comparison of existing TAD annotations

To date TAD discovery has been performed using Arrowhead and DomainCaller algorithms. The TADs called using Arrowhead were identified using relatively stringent criteria (identifying only TADs with strong intra-TAD interaction signal) resulting in the ~32% of the rice genome covered by TADs. TADs called using DomainCaller covered much higher proportion of the genome, but the intra-TAD signal tends to be much weaker (Supplementary Table 1).

TAD discovery in the three datasets using Armatus

Arrowhead and DomainCaller methods are among the oldest developed and have been superseded by newer algorithms. We have therefore decided to repeat TAD discovery in all three datasets using Armatus, which was shown to discover TADs with high intra-TAD interaction frequencies[4]. The size and number of domains called by Armatus depends on a single parameter γ (Supplementary Table 2). To optimize γ we used the D2 dataset (replicate 1) which had an intermediate number of valid interactions as evaluated by HiC-Pro (Supplementary Table 3). We tried three values (γ=0.3, 0.4, 0.5). We pre-filtered the calls with minimum TAD size of 20kb (4x bin size). Filtering follows from the statement by the algorithm authors that domains consisting of just one or two fragments do not capture higher-order spatial relationships (e.g. triad closure) and interaction frequencies between adjacent fragments are likely large by chance[4]. We used the total number of domains identified, domain size and visual inspection to choose the optimal gamma value (Supplementary Table 2). The main difference between γ=0.3 and γ=0.4 was that some of the smaller domains were fused, which is both consistent with the effect of decreasing γ described by the authors and with postulated hierarchical structure of rice topological domains[4].

We then used Armatus (γ=0.4) for TAD discovery using contact maps produced by HiC-Pro for all three datasets. First, we checked for concordance in TAD calls between replicates. In general, concordance in TAD boundary calls between replicates has been shown to be quite low[5]. We have observed similar pattern in our data with Jaccard indexes of 0.24, 0.45 and 0.35 for D1, D2 and D3 data respectively. Overall D2 had the highest concordance between replicates. We than merged the replicates and performed TAD calling again (Armatus, γ=0.4). We evaluated the resulting TAD calls by comparing TAD size, total genome coverage by TADs and the within-TAD contact frequency. The within-TAD contact frequency is expected to be affected by the number of sequencing reads. We therefore compared the intra-TAD interactions not only using the datasets used to call TADs but also the other two datasets (Supplementary Table 3). As expected, the median values were lower for datasets with lower number of valid read pairs. D2 achieved the best balance between TAD size and intra-TAD interaction frequencies.

Taken together D2 had a sufficient number of reads to call TADs at 5kb resolution, best concordance between replicates and good balance between TAD size and strength of intra-TAD interactions. TADs called from D2 were therefore used for further analysis.

References

1. Dong, P. *et al.* 3D chromatin architecture of large plant genomes determined by local A/B compartments. *Mol Plant* **10**, 1497-1509 (2017).
2. Liu, C., Cheng, Y.J., Wang, J.W. & Weigel, D. Prominent topologically associated domains differentiate global chromatin packing in rice from Arabidopsis. *Nat Plants* **3**, 742-748 (2017).
3. Dong, Q. *et al.* Genome-wide Hi-C analysis reveals extensive hierarchical chromatin interactions in rice. *The Plant Journal* **94**, 1141-1156 (2018).
4. Filippova, D., Patro, R., Duggal, G. & Kingsford, C. Identification of alternative topological domains in chromatin. *Algorithms for Molecular Biology* **9**, 14 (2014).
5. Forcato, M. *et al.* Comparison of computational methods for Hi-C data analysis. *Nature Methods* **14**, 679-685 (2017).
